# A New Classification of Prodrugs: Regulatory Perspectives

**DOI:** 10.3390/ph2030077

**Published:** 2009-10-14

**Authors:** Kuei-Meng Wu

**Affiliations:** Center for Drug Evaluation and Research, Food and Drug Administration, 10903 New Hampshire Avenue, Silver Spring, MD 20993, USA; Email: kueimeng.wu@fda.hhs.gov; Tel.: +1 301-796-9883

**Keywords:** prodrug, drug development, prodrug classification

## Abstract

Many therapeutic agents are manufactured and administered in prodrug forms. In this paper, a new classification system for prodrugs is proposed to provide useful information about where in the body a prodrug is converted to the active drug. In this system, prodrugs are classified into Type I or Type II and the respective Subtypes IA, IB, IIA, IIB or IIC based on their sites of conversion into the final active drug form. For Type I prodrugs, conversion occurs intracellularly (*e.g.*, antiviral nucleoside analogs, lipid-lowering statins), whereas conversion of Type II prodrugs occurs extracellularly, for examples in digestive fluids, systemic circulation or other extracellular body fluids (*e.g.*, etoposide phosphate, valganciclovir, fosamprenavir). Type IA prodrugs refer to those that are converted at the cellular targets of therapeutic actions, whereas Type IB prodrugs’ conversion occurs in the primary metabolic tissues such as liver, gut, or lung. For Type II prodrugs, the conversion process could either take place extracellularly in the milieu of gastrointestinal fluids (Type IIA), in the systemic circulation and/or other systemic extracellular fluid compartments (Type IIB), or near therapeutical target cells (Type IIC). A prodrug may belong to multiple categories and be recognized as a Mixed-Type prodrug. For example a prodrug may be converted both in target cells and metabolic tissues such as liver (*i.e.*, named as a Type IA/IB prodrug), or one converted in both GI fluids and systemic circulations (*i.e.*, named as a Type IIA/IIB prodrug). The Mixed-Type compound can be further distinguished as a Parallel Mixed-Type or Sequential Mixed-Type prodrug depending on the conversion processes that proceed with, either in concurrent or in sequential steps. Because traditional analysis of drug actions has always been focused on the site of action and mode of action, the proposed classification of prodrugs based on cellular locations of conversion is in line with current thought processes of regulatory review and risk assessment of both prodrug and active drug. By gaining insights regarding the site of action through prodrug nomenclature, risk benefit evaluation can be made more efficiently because both information on kinetics and impact of tissues involved are adequately revealed through prodrug subtype designated. In conclusion, the new system of classification will add to existing knowledge of prodrug classifications, and will provide improved insight into the contributory roles of both prodrug and active drug in the product’s efficacy and safety, and their risk-benefit assessment.

## Introduction

A prodrug can be defined as a drug substance that is inactive in the intended pharmacological actions and is must to be converted into the pharmacologically active agent by metabolic or physico-chemical transformation. Prodrugs can exist naturally such as many phytochemicals/botanical constituents and endogenous substances, or they can result from synthetic or semisynthetic processes – produced intentionally as part of a rational drug design or unintentionally during drug development. Examples of prodrugs that exist naturally or were produced unintentionally during drug development include aspirin, psilocybin, parathion, irinotecan, codeine, heroin, L-dopa, and various antiviral nucleosides. Examples of products resulting from pharmaceutical processes as part of strategically targeted drug design include sulfasalazine, oseltamivir, various nonsteroidal anti-inflammatory drugs (ketoprofen, diclofenac), statins (lovastatin, simastatin), ACE inhibitors (captopril, lisinopril) and penicillin-related agents (bacampicillin, sarmoxicillin). 

The need to design and produce a prodrug is often related to issues such as (1) bioavailability, such as poor aqueous solubility (*e.g.*, corticosteroids), (2) poor absorption/permeability (*e.g.*, ampicillin), (3) high first pass extraction (*e.g.*, propranolol), (4) instability (*e.g.*, short half-life, such as dopamine), (5) poor site specificity (*i.e.*, that the site of action of an active drug is rather nonspecific such as anticancer agents), (6) incomplete absorption (epinephrine), (7) unfavorable organoleptic properties (chloramphenicol), (8) pharmaceutical formulation difficulties, and (9) other adverse effects or toxicities [1,2].

## Current Classifications of Prodrugs

There are potentially many methods of classifying prodrugs. These could include those: (1) based on therapeutic categories; for example, anticancer prodrugs, antiviral prodrugs, antibacterial prodrugs, nonsteroidal anti-inflammatory prodrugs, cardiovascular prodrugs, etc.; (2) based on the categories of chemical linkages or moiety/carriers that attach to the active drug; for example, esteric prodrugs, glycosidic prodrugs, bipartite prodrugs, tripartite prodrugs, and antibody-, gene-, virus-directed enzyme prodrugs; or (3) based on functional categories using strategic approaches to circumvent deficiencies inherent to the active drug; for example, prodrugs for improving site specificity, prodrugs to bypass high first-pass metabolism, prodrugs for improving absorption, and prodrugs for reducing adverse effects [3,4,5]. 

## A New Classification of Prodrugs

The primary goal in pharmaceutical design of a prodrug has been to circumvent some disadvantageous pharmacodynamic or pharmacokinetic property of the active drug; *e.g*., to increase bioavailability or to reduce adverse effects. However, principal concerns during prodrug product development are two-fold: (1) whether the prodrug converts sufficiently fast and completely into the active drug format (in other words how long and how much remains intact in the body); and (2) whether the prodrug contributes significantly to the active drug’s toxicity profile (which is especially important when it exhibits unique and different toxicities compared to the converted active drug). These concerns are interrelated and are closely associated with the strategic goal of improving a drug product’s quality, safety, and efficacy profiles. Thus, from the standpoint of assessing risk-benefit of a prodrug, a classification system based on the site of its conversion into the active drug form would be most useful because it can provide insight into the kinetics of the conversion process and the contributory role of prodrug and active drug to the product’s efficacy and safety. Under this proposal, prodrugs are classified into Type I and Type II, based on their cellular sites of conversion into the final active drug form, with Type I being those that are converted intracellularly (*e.g.*, anti-viral nucleoside analogs, lipid-lowering statins,), and Type II being those that are converted extracellularly, especially in digestive fluids or the systemic circulation (*e.g.*, etoposide phosphate, valganciclovir, fosamprenavir, antibody-, gene- or virus-directed enzyme prodrugs [ADEP/GDEP/VDEP] for chemotherapy or immunotherapy). Both types can be further categorized into Subtypes, *i.e.*, Type IA, IB and Type IIA, IIB, and IIC based on whether or not the intracellular converting location is also the site of therapeutic action, or the conversion occurs in the gastrointestinal (GI) fluids or systemic circulation (see Table 1).

Type IA prodrugs include many antimicrobial and chemotherapy agents (*e.g.*, 5-flurouracil). Type IB agents rely on metabolic enzymes, especially in hepatic cells, to convert the prodrugs intracellularly to active drugs. Type II prodrugs are converted extracelluarly, either in the milieu of GI fluids (Type IIA), within the systemic circulation and/or other extracellular fluid compartments (Type IIB), or near therapeutic target tissues/cells (Type IIC), relying on common enzymes such as esterases and phosphatases or target directed enzymes. Importantly, prodrugs can belong to multiple subtypes (i.e., Mixed-Type). A Mixed-Type prodrug is one that is converted at multiple sites, either in parallel or sequential steps. For example, a prodrug, which is converted concurrently in both target cells and metabolic tissues, could be designated as a “Type IA/IB” prodrug (e.g., HMG Co-A reductase inhibitors and some chemotherapy agents; note the symbol “ / ” applied here). When a prodrug is converted sequentially, for example initially in GI fluids then systemically within the target cells, it is designated as a “Type IIA-IA” prodrug (*e.g.*, tenofovir disoproxil fumarate; note the symbol “ - ” applied here). Many ADEPs, VDEPs, GDEPs and futuristic nanoparticle- or nanocarrier-linked drug moieties can understandably be Sequential Mixed-Type prodrugs. To differentiate these two Subtypes, the symbol dash “ - ” is used to designate and to indicate sequential steps of conversion, and is meant to distinguish from the symbol slash “ / ” used for the Parallel Mixed-Type prodrugs.

Because traditional analysis of drug actions has always been focused on the *site* of action and *mode* of action, the proposed classification of prodrugs based on cellular locations of conversion is in line with current thought processes of regulatory review and risk assessment of both prodrug and active drug. For example, a Type IIA prodrug would indicate that it is converted into active drug in the GI fluids, and that the safety/toxicity profile of the drug product can be fully reflected by, and interpreted in lieu of the active drug (assuming that the conversion is complete, as validated by the fact that there is no unconverted prodrug left at the GI site and there is no measurable systemic prodrug). More detailed discussions on, and analytical approaches to, risk assessment of prodrugs can be found in the article published previously [7].

**Table 1:** Classification of Prodrugs.

Prodrug Types	Site of Conversion	Subtypes	Tissue Location of Conversion	Examples
**Type I**	Intracellular	A	Therapeutic Target Tissues/Cells	**Type IA:** Acyclovir 5-Flurouracil Cyclophosphamide Diethlstilbestrol diphosphate L-Dopa 6-Mercaptopurine Mitomycine C Zidovudine
		B	Metabolic Tissues (liver, GI mucosal cell, lung, etc.)	**Type IB:** Cabamazepine Captopril Carisoprodol Heroin Molsidomine Paliperidone Phenacetin Primidone Psilocybin Suldinac Tetrahydrofurfuryl disulfide
**Type II**	Extracellular	A	GI Fluids	**Type IIA:** Lisdexamfetamine Loperamide oxide Oxyphenisatin Sulfasalazine
		B	Systemic Circulation and Other Extracellular Fluid Compartments	**Type IIB:** Acetylsalicylate Bacampicillin Bambuterol Chloramphenicol succinate Dihydropyridine pralixoxime Dipivefrin Fosphenytoin
		C	Therapeutic Target Tissues/Cells	**Type IIC:** ADEPs GDEPs VDEPs

In summary, by gaining insights through the proposed nomenclature, risk-benefit assessment can be made more effectively because information related to kinetics and the impact of target and metabolic tissues are adequately revealed by the prodrug type designated.

## Conclusions

From a regulatory perspective, a new classification system of prodrugs is proposed with two major types and 2–3 respective subtypes described (*i.e.*, Types IA, IB, IIA, IIB and IIC). This new classification system of prodrugs can help in the understanding of a drug product’s pharmacokinetics, safety and efficacy. It provides a more systematic approach to categorizing a prodrug based on the biological site of conversion. This new system of classification will add to existing knowledge of prodrug classifications and will facilitate risk-benefit assessment processes during product development of a prodrug.
